# The Involvement of OsPHO1;1 in the Regulation of Iron Transport Through Integration of Phosphate and Zinc Deficiency Signaling

**DOI:** 10.3389/fpls.2016.00396

**Published:** 2016-04-06

**Authors:** Chorpet Saenchai, Nadia Bouain, Mushtak Kisko, Chanakan Prom-u-thai, Patrick Doumas, Hatem Rouached

**Affiliations:** ^1^Biochimie et Physiologie Moléculaire des Plantes Research Unit, Institut National de la Recherche Agronomique – Centre National de la Recherche Scientifique – Montpellier UniversityMontpellier, France; ^2^Agronomy Division, Department of Plant and Soil Sciences, Faculty of Agriculture, Chiang Mai UniversityChiang Mai, Thailand

**Keywords:** ions homeostasis, iron, phosphate, rice, signaling crosstalk, zinc

## Abstract

Plants survival depends on their ability to cope with multiple nutrient stresses that often occur simultaneously, such as the limited availability of essential elements inorganic phosphate (Pi), zinc (Zn), and iron (Fe). Previous research has provided information on the genes involved in efforts by plants to maintain homeostasis when a single nutrient (Pi, Zn, or Fe) is depleted. Recent findings on nutritional stress suggest that plant growth capacity is influenced by a complex tripartite interaction between Pi, Zn, and Fe homeostasis. However, despite its importance, how plants integrate multiple nutritional stimuli into complex developmental programs, and which genes are involved in this tripartite (Pi ZnFe) interaction is still not clear. The aim of this study was to examine the physiological and molecular responses of rice (*Oriza sativa L.*) to a combination of Pi, Zn, and/or Fe deficiency stress conditions. Results showed that Fe deficiency had the most drastic single-nutrient effect on biomass, while the Zn deficiency-effect depended on the presence of Pi in the medium. Interestingly, the observed negative effect of Fe starvation was alleviated by concomitant Pi or PiZn depletion. Members of the *OsPHO1* family showed a differential transcriptional regulation in response PiZnFe combinatory stress conditions. Particularly, the transcripts of the *OsPHO1;1* sense and its natural antisense *cis-NatPHO1;1* showed the highest accumulation under PiZn deficiency. In this condition, the *Ospho1;1* mutants showed over-accumulation of Fe in roots compared to wild type plants. These data reveal coordination between pathways involved in Fe transport and PiZn signaling in rice which involves the *OsPHO1; 1*, and support the hypothesis of a genetic basis for Pi, Zn, and Fe signaling interactions in plants.

## Introduction

Phosphorus (P) is an essential nutrient for optimal plant growth, development, and productivity. The effect of inorganic phosphate (Pi) deficiency on crop yield has become a worldwide concern in recent years due to the pressing problem of food availability (Holford, 1997; Abelson, 1999; Neset and Cordell, 2012). Accordingly, this issue has received increasing attention. To date, research has provided a comprehensive view of the adaptive strategies used by plants to cope with Pi deficiency (Rouached et al., 2010a; Thibaud et al., 2010; Nussaume et al., 2011; Lin et al., 2014). Lists of genes differentially expressed in Pi deficient conditions compared to control conditions have been produced, and signaling pathways involved in maintaining the homeostasis of Pi have been proposed (Misson et al., 2005; Lin et al., 2008, 2014). However, the practical application of such knowledge is hindered by complex crosstalk linking Pi nutrition and nutrition of other essential micronutrients, e.g., elements are not assimilated, but are taken up zinc (Zn) and iron (Fe) (Huang et al., 2000; Bouain et al., 2014a; Khan et al., 2014). Such interconnections may account for the shortcomings of current agronomic models that typically focus on improving the assimilation of individual elements (Rouached et al., 2011a; Ova et al., 2015). Yet despite their fundamental importance, the molecular bases, biological significance, and agronomical repercussions of these interactions remain unknown.

Our current understanding of genes involved in the transport of Pi into and within the plant has considerably progressed in recent decades (Poirier and Bucher, 2002; Bayle et al., 2011; Nussaume et al., 2011; Sun et al., 2012; Poirier and Jung, 2015). The first step of Pi transport in plants is its acquisition at the soil-root interface. Genes belonging to the *PHT1* family, which are involved in Pi uptake and transport, have been cloned, and characterized in both monocotyledons and dicotyledons (Nussaume et al., 2011; Sun et al., 2012). The increased interest in genes involved in the second rate-limiting step in Pi transport within the plant, i.e., its transfer to xylem vessels, led to the identification of *PHO1* gene in *Arabidopsis* as a key player in this process (Hamburger et al., 2002; Secco et al., 2010). *Atpho1* mutants showed defects in Pi homeostasis in the form a marked reduction in Pi transfer from the roots to the shoot, which was accompanied by low-shoot and high-root Pi concentrations (Hamburger et al., 2002; Rouached et al., 2011b; Poirier and Jung, 2015). The PHO1 protein feature is characterized by the presence of two distinct domains, named SPX and EXS, which may play a role in either Pi transport/ sensing and sorting proteins to endomembranes, respectively (Wang et al., 2004; Wege et al., 2016). Thus, the PHO1 protein does not share protein homology with any other characterized Pi transporters (Wang et al., 2004). The *Arabidopsis* genome contains 11 members of the PHO1 family (Wang et al., 2004). So far, only three members, AtPHO1, AtPHO1;H1 (Stefanovic et al., 2007) and AtPHO1;H3 (Khan et al., 2014), have been reported to be involved in Pi root-to-shoot translocation. These data indicate that the role of members of the *Arabidopsis* PHO1 family is not restricted to Pi transport and homeostasis. For example, the *PHO1;H4* homolog is involved in the response of hypocotyls to blue light (Kang and Ni, 2006), as well as in seed size (Zhou et al., 2009) and flowering (Zhou and Ni, 2009). The role of the *PHO1* gene family has been investigated in a few crop species, including rice (*Oryza sativa L.*) (Secco et al., 2010). Three rice *PHO1* genes, named *OsPHO1;1*, *OsPHO1;2*, and *OsPHO1;3* have been identified. So far, only *OsPHO1;2* has been shown to play a key role in the transfer of Pi from roots to shoots and regulated by Pi deficiency (Secco et al., 2010). The role of the other two rice *PHO1* genes, *OsPHO1;1*, and *OsPHO1;3*, remains to be investigated.

Pi is known to interact with many micronutrients including Zn and Fe (for a review, see Bouain et al., 2014a; Briat et al., 2015). The effects of Pi, Zn, or Fe availability on plant physiology and transcriptomic have typically been studied individually (Misson et al., 2005; Van De Mortel et al., 2006; Zheng et al., 2009). Although this is an important way to address the question, it can only provide a partial view of the situation. The interactions between Pi, Zn, and Fe metabolisms have been demonstrated both in graminaceous and non-graminaceous plants (Zheng et al., 2009; Briat et al., 2015). These interactions have started being documented at a molecular level, showing that Fe or Zn deficiency modifies the expression of genes involved in Pi transport and assimilation, and vice-versa (Zheng et al., 2009; Briat et al., 2015). Nevertheless, the characterization of these interactions is still in its infancy. In addition to the well-established antagonistic relationship between Fe and Zn in plants (Haydon et al., 2012; Shanmugam et al., 2012; Briat et al., 2015), the existence of an interaction between the homeostasis of Pi and Zn (Huang et al., 2000; Bouain et al., 2014b; Khan et al., 2014; Ova et al., 2015) and Pi and Fe (Zheng et al., 2009) was recently reported, pointing to the existence of a complex tripartite PZnFe signaling interaction to regulate plant growth. Therefore, undertaking an integrative study to clarify the exact molecular mechanisms that coordinate the Pi, Fe, and Zn deficiency pathways and thus shape the plants development during multiple PZnFe deficiency stress is of great interest.

Rice (*Oryza sativa L.*) is one of the most important cereal crops for human consumption, and feeds about half the world population^1^. The effect of individual Pi, Zn, or Fe deficiencies on rice shoot and root growth is fairly well documented (e.g., Wissuwa et al., 2006; Zheng et al., 2009; Secco et al., 2010), but the effect of their combined stresses on plant biomass has been the subject of fewer investigations. In this context, the primary aim of the present study was to broaden our understanding of the long-term effect of different combinations of stresses caused by a deficiency in these essential macro- and micronutrients in rice. Wild type plants and selected mutant lines affected in members of *OsPHO1* gene family, namely *Ospho1;1*, were included in this work. Pi, Zn, and Fe ion concentrations and biomass were determined in rice plants grown in seven different combinations of growing conditions. Physiological and gene expression analyses revealed the involvement of *OsPHO1;1* in the interaction between Fe transport and PiZn signaling pathways in rice.

## Results

### Effects of a Combination of Stresses Caused by Pi, Zn, and Fe Deficiency on Biomass in Rice (Nipponbare)

**FIGURE 1:**
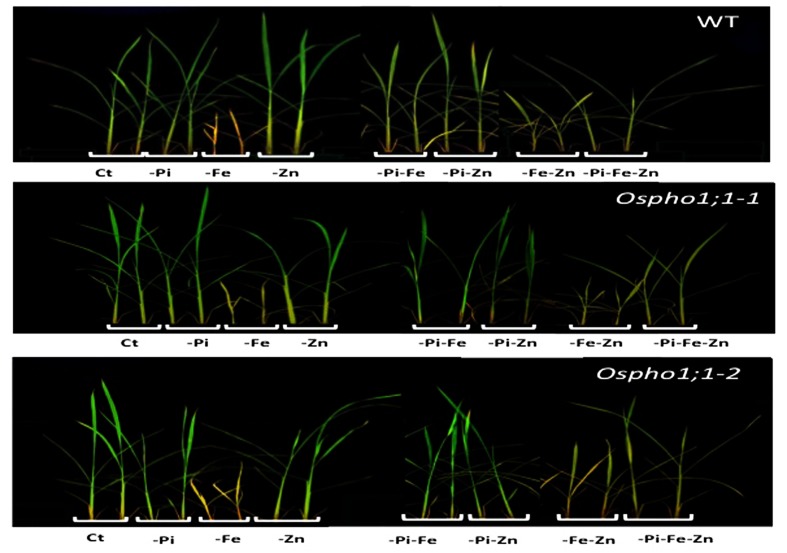
**Phenotypes of wild type (WT) rice plant and *Ospho1;1-1* and *Ospho1;1-2* mutant lines grown under individual and combinatory ion deficiency stresses.** Seedlings were grown for 21 days on Yoshida medium with different ionic concentrations: Complete Yoshida media (Ct) or lacking either individual phosphate (-Pi), zinc (Zn), or iron (Fe); double elements -Pi-Zn, -Pi-Fe, -Zn-Fe, or triple elements -Pi-Fe-Zn. Photographs were taken at 21 days old. The experiment was repeated three times.

**FIGURE 2:**
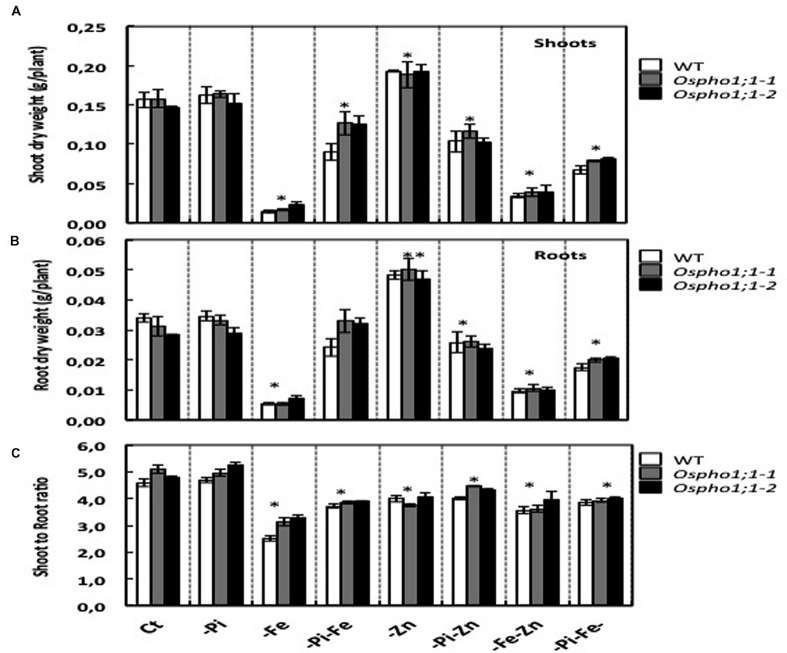
**Biomass of wild typerice plant and *Ospho1;1-1* and *Ospho1;1-2* mutant lines grown on different mineral element supply.** **(A)** Wild type (WT), **(B)** *Ospho1;1-1*, and **(C)** *Ospho1;1-2* mutant seedlings were grown on Yoshida medium with different ionic concentrations: Complete medium (Ct) or lacking either individual element phosphate (-Pi), zinc (Zn), or iron (Fe); double elements -Pi-Zn, -Pi-Fe, -Zn-Fe, or triple elements -Pi-Fe-Zn. FW; Fresh weight is expressed as g/plant. DW, Dry weight. The experiment was repeated three times. The data are given as means ± SE (*n* = 9). Asterisk indicates statistical significance, *P* < 0.05. Double asterisk indicates statistical significance, *P* < 0.01.

Wild type rice plants (variety Nipponbare) were grown hydroponically in seven different growing conditions including single (-Pi, -Zn, and -Fe), double (-Pi–Fe, -Pi–Zn, and -Zn–Fe), and triple (-Pi–Fe–Zn) ion deficiency stresses. **Figure 1** shows the phenotypes of 21-days old wild type rice plants. The most severe effect on shoot growth was observed for Fe deficiency in comparison to the effects of single Pi or Zn deficiency stress. Interestingly, the Fe deficiency stress was alleviated when combined with Pi limitation (-Pi-Fe or –Pi-Fe-Zn) (**Figure 1**). This is reminiscent of data showing that the striking aspect of the inhibition of primary root elongation in *Arabidopsis* was recovered when the concentrations of Fe and Pi in medium were simultaneously reduced (Ward et al., 2008). This also suggests that manipulating Pi availability to a rice plant could be a valuable strategy for improving a plant’s ability to tolerate Fe deficiency. To gain quantitative insights into these combined stresses effect, the rice biomass was assessed by measuring the roots and shoots dry weight (**Figures 2A,B**). Stress caused by Fe deficiency had the strongest negative influence on the shoot and root dry biomass, whereas Zn deficiency had a slight yet significant positive effect (**Figures 2A,B**). Pi limitation alone had no effect on these parameters. Change in Pi together with changes in Fe or Zn had striking effects. Simultaneous Pi and Fe stress (PiFe) alleviated the severe stress caused by the Fe deficiency alone, while stress caused by a simultaneous deficiency in Pi and Zn had a negative effect. Double (FeZn) or triple (PiFeZn) deficiencies had a negative effect on biomass similar to the effect of Fe deficiency alone. Shoot to root dry weight ratio showed that except for -Pi stress, all other nutrient stress conditions tested had a negative effect on biomass (**Figure 2C**).

Taken together, the results indicate that supply of one mineral element has a sometimes strong effect on the plant response to supply of other elements. For example, as shown in **Figure 1**, Fe deficient plants were smaller and chlorotic, but Fe deficient plants also lacking P and Zn looked healthier in comparison. These data underline thus the importance of the coordination between Pi, Zn, and Fe homeostasis in the control of rice growth capacity and shoot and root biomass.

### Effects of a Combination of Nutrient Availabilities on Pi, Zn, and Fe Concentrations in Rice (Nipponbare)

**FIGURE 3:**
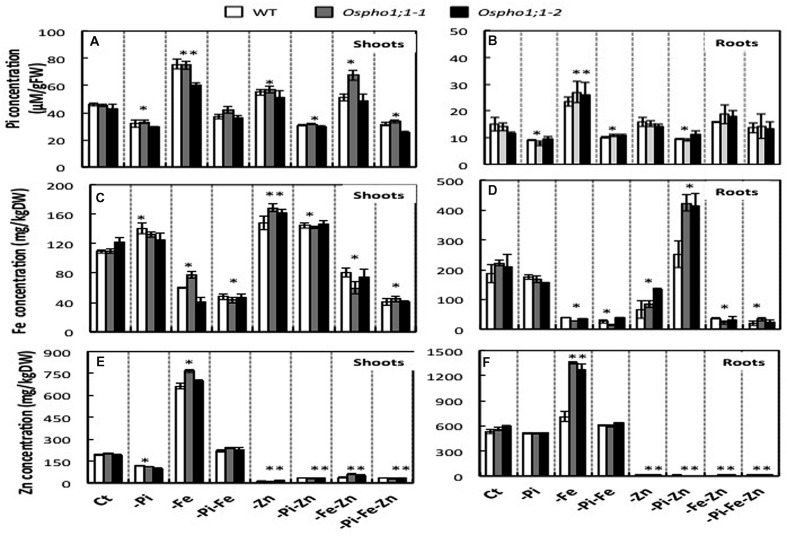
**Phosphate, Zinc, and Iron concentrations in wild type (WT) rice and *Ospho1;1* mutant lines plant grown under individual and combinatory ions deficiency stresses.** **(A,C,E)** Present Pi, Zn, and Fe concentrations in the shoots, respectively. **(B,D,F)** Present Pi, Zn, and Fe concentrations in the roots, respectively. After a 10 days of germination in 1/4 full-strength of Yoshida media, plants were grown for 3 weeks on full-strength Yoshida medium with different ionic concentrations: Complete medium (Ct) or lacking either individual element Pi, Zn, or Fe; double elements PiZn, PiFe, ZnFe, or triple elements PiFeZn. Shoots and roots were harvested separately and the Pi and Zn and Fe were extracted and their concentrations were determined as indicated in material and methods. Pi concentrations are calculated per fresh weight. Zn and Fe concentrations are calculated per dry weight. The experiment was repeated three times. The data are given as means ± SE (*n* = 9). Asterisk indicates statistical significance, *P* < 0.05. Double asterisk indicates statistical significance, *P* < 0.01.

The effects of a combined Pi, Zn, and/or Fe deficiency stress on the accumulation of each of these elements in rice were assessed in wild type rice plants grown for 21 days on complete solution or in solution deficient in Pi, Fe, or Zn, and combinations of two and all three deficiencies. The concentration of each element was determined both in shoot and roots tissues as indicated in material section. Under Pi deficiency, the Pi concentration was reduced by ≈1/4-fold compared to rice plants grown on complete medium composition, thereby confirming the plants suffered from Pi deficiency. A comparable effect (≈1/4-fold decrease) was observed in response to PiFe, PiZn double stresses or PiZnFe triple deficiency stresses. Pi concentrations the shoot increase by 1.5-fold in response Fe deficiency, but only by ≈0.5 by Zn or FeZn deficiency. The Zn concentrations in the shoots and in the roots in wild type rice plants subjected to the seven conditions detailed above decreased considerable in all conditions in which Zn was omitted. Zn concentration in the shoot was reduced by Pi deficiency stress, whereas combined Pi and Fe deficiency had no effect. However, the individual Fe deficiency considerable increased Zn content in both the shoot (>threefold increase) and to a lesser extent in the roots (>1.5-fold increase). Like Zn, the Fe content in the shoot and roots of rice plants subjected to nutritional deficiency was strongly reduced when this element was omitted. Fe content increased slightly in the shoots of Pi, Zn, or Pi/Zn deficient plants (**Figure 3**). A striking increase in Fe content was observed in the roots of plants under combined -Pi-Zn stress, which was not observed in any case of single stress. This clearly demonstrates that Pi and Zn homeostasis not only interact but also influence Fe nutrition.

### Effects of Pi, Zn, and Fe Availability on the Expression of Members of the *OsPHO1* Gene Family

**FIGURE 4:**
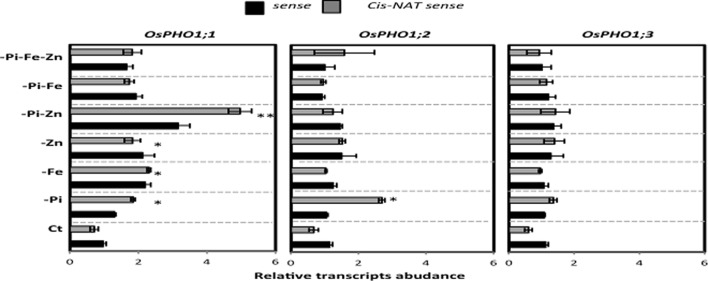
**Transcripts abundance of sense and anti-sense of *OsPHO1;1* and *OsPHO1;2* in *OsPHO1;3* as determined by qRT-PCR.** *OsActin1* was used as an internal reference. qRT quantitative real-time-PCR was performed to analyze the *OsPHO1;1* and *OsPHO1;2* in *OsPHO1;3* mRNA levels in response to under multiple nutritional stresses: complete Yoshida medium (Ct) or lacking to either individual element phosphate (-Pi), zinc (-Zn), or iron (-Fe); double elements -Pi–Zn, -Pi–Fe, -Zn–Fe, or triple elements -Pi-Fe-Zn. All values are relative to wild-type (WT) roots. The experiment was repeated three times. The data are given as means ± SE (*n* = 9). Asterisk indicates statistical significance, *P* < 0.05. Double asterisk indicates statistical significance, *P* < 0.01.

The previous ion concentrations analysis showed that rice plants exposed to either Fe or Zn deficiency or to FeZn simultaneous stress overaccumulated Pi in the shoot (**Figure 3**). Previous research work by Secco et al. (2010) suggests that members of the OsPHO1 gene family (*OsPHO1;1*, *OsPHO1;2*, and *OsPHO1;3*) are good candidates for the transfer of Pi from the roots to the shoot. Each of these three *OsPHO1* genes has its own *cis*-natural antisense transcript (*cis*-NATs) (Secco et al., 2010), which could play a role by stabilizing the transfer of its sense gene, as demonstrated by Jabnoune et al. (2013) for *OsPHO1;2*. In the present study, the regulation of the sense and antisense transcripts of the *OsPHO1* gene family member was analyzed in wild type rice roots subjected to the each of the above-mentioned growing ions-deficient conditions. Results in **Figure 4** show that in most cases, there was an increase in sense or antisense transcript level for the three *OsPHO1* gene family members in response to Pi deficiency. Previous results by Secco et al., (2010) showed the most abundantly expressed gene was OsPHO1;2 in the roots, for both sense and antisense transcripts. Our results revealed an interesting profile was observed for *OsPHO1;1* gene in response to the stress caused by combined PiZn deficiency (**Figure 4**). It is worth noting that the high antisense:sense ratio of *OsPHO1;1* observed under this condition was not observed when rice plants were treated with single stress caused by Pi or Zn deficiency. This result suggests that the expression of *OsPHO1;1* depends on simultaneous PiZn deficiency signaling, and raise the questions on the involvement of the *cis*-natural antisense its regulation, in similar manner to OsPHO1;2 (Secco et al., 2010).

### Effects of a Combination of Pi, Zn, and Fe Deficiency Stresses on the Production of Biomass and the Concentration of Ions in the *Ospho1;1* Rice Mutant

The role of *OsPHO1;1* in rice is not known to date. The over-concentration of its transcripts in the combined Pi/Zn deficiency stress prompted us to assess the effect the mutation of *OsPHO1;1* in shoot and root biomass when grown in the above-mentioned combinations of nutrient stresses. Two independent lines, named *Ospho1;1-1* and *Ospho1;1-2* were used for in this analysis (Secco et al., 2010). **Figure 1** shows the phenotypes of 21 days old rice *Ospho1;1* mutant lines grown on different medium compositions. The results presented in **Figures 1** and **2** for the two *Ospho1;1* mutant lines were comparable to the wild type in term of growth and biomass. Nevertheless, analysis of the concentration of ions (Pi, Zn, and Fe) (**Figures 3A–F**) in rice tissues revealed an over-accumulation of Fe (twofold) in the roots of *Ospho1;1* mutants compared to wild type plant. This difference in Fe concentration in roots was not observed in the shoots of the rice plants (**Figures 3E,F**). These data show that the regulation of Fe content root was altered in the *Ospho1;1* mutant lines in response to the double PiZn deficiency, supporting a role for *OsPHO1;1* in the regulation of Fe homeostasis.

## Discussion

Compared with a growing list of documented effects of individual nutritional stress in plants, little is known about the effects of combinations of macro- and micronutrient deficiencies in plants (Mathan and Amberger, 1977; Hirsch et al., 2006; Bournier et al., 2013; Jain et al., 2013; Bouain et al., 2014a; Briat et al., 2015; Rai et al., 2015). To date research on Pi, Zn, and Fe nutrition has focused on the regulation of the homeostasis of each element individually. The effect of one element on the concentration of the other one is just starting to be documented, and point to the existence of a complex tripartite interaction between these three nutrients (Huang et al., 2000; Zheng et al., 2009; Bouain et al., 2014a; Briat et al., 2015; Ova et al., 2015). But despite its importance, our understanding of how the signaling pathways of these nutrients are “wired” into functional networks to control plant growth is still very limited.

The antagonistic relationship between Fe and Zn in plants on one hand, and between Zn or Fe starvation and Pi concentration on the other hand has been documented in many plant species (Huang et al., 2000; Zheng et al., 2009; Bouain et al., 2014b; Khan et al., 2014; Briat et al., 2015). The present study provides additional evidence for the existence and for the importance of two-by-two interactions between these ions in rice. For example, the observed effects of Pi or Zn deficiency on root biomass were only visible when the rice plants were subjected to double PiZn deficiency. Interestingly, in this condition, the reduction in root biomass occurred concomitantly with a marked increase in the concentration of Fe. The consequence of the accumulation of Fe on plant development has been described in *Arabidopsis* (Ward et al., 2008). These authors proposed that Fe excess in Pi deficient *Arabidopsis* plants, grown in agar plates, was the cause of a severe reduction in the primary root growth. Recently, it has been proposed that iron-dependent callose deposition adjusts root meristem maintenance to Pi availability (Muller et al., 2015). Consistently, simultaneous Pi and Fe deficiency (PiFe) restored primary root growth. In the present study, while the Fe deficiency had the biggest single-nutrient effects in rice plants grown hydroponically, this effect appeared to depend on the presence of Pi, or Zn, or of both elements simultaneously, in the medium (**Figure 1**). Indeed, stress caused by the simultaneous deficiency of two nutrients, namely PiFe or ZnFe, alleviates the effect of single Fe deficiency (Fe) on biomass, a phenomenon that was even clearer under triple stress (PiZnFe) (**Figure 1**). Thus, Fe deficiency cannot be considered as being solely responsible for the observed reduction in growth, but rather the interaction between the homeostasis of the three nutrients PiZnFe. Together, the data presented in this study shed light on the existence of a tripartite interaction between these macro-and micronutrients in plants and provide the basis for investigating how plants integrate multiple nutrient deficiencies signaling in complex plant developmental programs.

Plants must adapt to a fluctuating environment, and changes in gene expression are a key part of this adaptation. The identification of key genes involved in the co-regulation of micro- and macronutrient homeostasis has become the focus of major interest (Zheng et al., 2009; Rouached et al., 2010b; Kellermeier et al., 2014; Khan et al., 2014). Recently, a member of the AtPHO1 gene family, *AtPHO1;H3*, was identified as an important player in the crosstalk between Zn deficiency signaling and the regulation of Pi homeostasis in *Arabidopsis* (Khan et al., 2014). In the present work, the regulation of the expression of a member of *OsPHO1* gene family, *OsPHO1;1*, also appears to depend on simultaneous PiZn deficiency signaling, and mutations of *Ospho1;1* lead to over-accumulation of Fe mainly in roots compared to wild type plants. An attractive hypothesis to explain the results of this work would be that the effect of Ospho1.1 mutation interfere with the transport of Fe in roots under simultaneous -Pi-Zn deficiency. This hypothesis is supported by observations in yeast (*S. cerevisiae*) where the high-affinity Pi transporter PHO84 can regulate or transport metal ions such as “Metal-HPO4” (Luk et al., 2003a,b). Plants like *Arabidopsis* and rice possess various Pi transporters with significant similarity to PHO84, such as members of the *PHO1* gene family (e.g., *OsPHO1;1*). There is currently no evidence for either Fe or Fe/Pi transport by a plant Pi transporter, though this remains an intriguing possibility. The regulation of Os*PHO1;1* by its *cis-NATPHO1;1* can be also proposed for examination in light of the following arguments. First, the natural antisense of *OsPHO1;1* (*cis-NATPHO1;1*) was up-regulated by the combined -Pi-Zn deficiency stress (**Figure 4**). Second, a recent study by Jabnoune et al. (2013) reported an unexpected role for *cis-NATPHO1;2* in promoting *OsPHO1;2* translation and affecting Pi homeostasis and plant fitness. Considering this recent discovery (Jabnoune et al., 2013) and the results of the present study, one can hypothesize that *OsPHO1;1* could be a target in the -Pi-Zn deficiency signaling pathway and involved in the regulation of the Fe homeostasis.

## Conclusion

This study is the first documentation of the effect multiple nutritional stresses on rice growth and biomass, which is illustrated through the interaction between the homeostasis of essential macro(Pi) – and micronutrients (Zn and Fe). This obtained data provides evidence for the existence of a genetic basis for this tripartite nutrient PiZnFe interaction in plant. A biological function for the OsPHO1;1 in this tripartite interaction is proposed, and particularly in the regulation of Fe transport through integration of Pi and Zn deficiency signaling.

## Materials and Methods

### Plant Growth Conditions

The rice (*Oryza sativa* cv Nipponbare), wild type (WT) and mutant lines: O*spho1; 1-1* and *Ospho1;1-2*, was grown hydroponically under non-sterile conditions. Seeds were soaked in deionized water over night in dark then transferred to 1/4 full-strength Yoshida media for 10 days (Yoshida et al., 1976). Seedlings were transferred to the following modified Yoshida nutrient solution: 1.43 mM NH_4_NO_3_; 1.64 mM MgSO_4_; 0.75 mM CaCl_2_; 0.51 mM K_2_SO_4_; 0.33 mM NaH_2_PO_4_; 0.02 mM H_3_BO_3_; 0.01 mM MnCl_2_; 0.04 mM Fe-NaEDTA; 2.5 μM ZnSO_4_; 0.16 μM CuSO_4_; 0.08 μM (NH_4_)_6_Mo_7_O_24_; 2.5 μM MES buffer, adjust to pH 5.5. The nutrient solution was renewed every 5 days. Plants were grown in a growth chamber under the following environmental conditions: light/dark cycle of 14/10 h, temperature of 28/25°C, and RH of 80%. For the control condition, plants were kept in nutrient solution with the above-mentioned composition. The treatments were applied to 10 day-old plants. The concentration of individual nutrients in the complete media were 0.3 mM P, 0.025 mM Zn, and 0.04 mM Fe and zero in the deficient media for -Pi, -Zn, -Fe for single, double or triple ion deficiency combinations. The daytime temperature was 28°C and the night temperature 25°C with a 14/10 h light/dark regime. Plants were harvested at 3 weeks after transferred to treatments. Roots and shoots were separated, and immediately frozen in liquid N, and stored at -80°C.

### Real-Time Quantitative Reverse-Transcription PCR

DNA-free total RNA extraction from frozen root tissues was performed using Plant RNeasy extraction kit and RNAse-free DNAseI (SIGMA-ALDRCH, St Louis, MO, USA). Two micrograms of total RNA were reverse transcribed using Thermo^TM^ script RT (Invitrogen) according to the manufacturer’s protocol. Complementary DNA (cDNA) was used for real-time reverse-transcription PCR, which was performed with (LightCycler^®^480; Roche Diagnostics). PCR reactions containing 12.5 μL of the LightCycler^®^480 SYBR Green I Master mix (Roch, IN, USA), each of the forward and reverse primers, and 5 μL of a 1:50 cDNA dilution in a final volume of 25 μL were considered for gene expression analysis. All PCR reactions were performed in triplicate. In addition to the three *OsPHO1* genes, *OsActin1* gene was considered for the standardization of real-time PCR data. These genes were chosen based on previous analyses (Secco et al., 2010). Quantification of the relative transcripts levels was performed using the comparative CT method (Livak and Schmittgen, 2001; Rouached et al., 2008).

### Zinc and Iron Measurements

Shoots and roots material were dried at 72°C for at least 7 days. The dried tissues were ground and subjected (∼20 mg) to acid hydrolysis. Zn and Fe concentrations in the samples were determined using the Agilent 4200 Microwave Plasma-Atomic Emission Spectrometer (MP-AES 4200, Agilent, USA).

### Pi Measurements

For Pi extractions, weighed fresh shoots and roots were collected separately and incubated material in water for 3 h at 70°C. Pi measurements were performed as described by Rouached et al. (2010a). The quantification of Pi was performed by the molybdate assay according to Ames (1966).

### Statistical analysis

We carried out statistical analysis using The Analysis Toolpak add-in program for Excel for Macintosh (Microsoft Incorporated, USA). For all the t test analyses the difference is considered statistically significant with a probability of *p* < 0.05.

## Author Contributions

HR conceived and designed the experiment. CS, NB, and MK performing collected the experimental data. CS and NB equally contributed to this work (co-first authors). CP, PD, and HR analyzed data. HR wrote the manuscript. CP and PD assisted with the interpretation of the results and provided editorial support for the manuscript. All authors have read, edited, and approved the current version of the manuscript.

## Conflict of Interest Statement

The authors declare that the research was conducted in the absence of any commercial or financial relationships that could be construed as a potential conflict of interest.

## Footnotes

1 http://faostat3.fao.org/browse/Q/QC/E
